# Neoadjuvant immune checkpoint inhibitors in resectable non-small-cell lung cancer: a systematic review

**DOI:** 10.1016/j.esmoop.2021.100244

**Published:** 2021-08-31

**Authors:** E.B. Ulas, C. Dickhoff, F.L. Schneiders, S. Senan, I. Bahce

**Affiliations:** 1 Department of Pulmonology, Amsterdam University Medical Center, VU University Medical Center, Cancer Center Amsterdam, Amsterdam, the Netherlands; 2 Department of Surgery and Cardiothoracic Surgery, Amsterdam University Medical Center, VU University Medical Center, Cancer Center Amsterdam, Amsterdam, the Netherlands; 3 Department of Radiation Oncology, Amsterdam University Medical Center, VU University Medical Center, Cancer Center Amsterdam, Amsterdam, the Netherlands

**Keywords:** neoadjuvant, immune checkpoint inhibitors, immunotherapy, non-small-cell lung cancer, lung resection

## Abstract

**Background:**

The neoadjuvant use of immune checkpoint inhibitors (ICIs) in resectable non-small-cell lung cancer (NSCLC) is currently an area of active ongoing research. The place of neoadjuvant ICIs in the treatment guidelines needs to be determined. We carried out a systematic review of published data on neoadjuvant ICIs in resectable NSCLC to study its efficacy and safety.

**Patients and methods:**

A literature search was carried out using the MEDLINE (PubMed) and Embase databases to retrieve articles and conference abstracts of clinical trials measuring the efficacy [major pathological response (MPR) and pathological complete response (pCR)] and safety (failure to undergo resection, surgical delay, treatment-related adverse events (trAEs) grade ≥3) of neoadjuvant immunotherapy in resectable NSCLC until July 2021.

**Results:**

Nineteen studies with a total of 1066 patients were included in this systematic review. Neoadjuvant immunotherapy was associated with improved pathological response rates, especially in combination with chemotherapy. Using mono ICI, dual therapy–ICI, chemoradiation–ICI, radiotherapy–ICI, and chemo–ICI, the MPR rates were 0%-45%, 50%, 73%, 53%, and 27%-86%, respectively. Regarding pCR, the rates were 7%-16%, 33%-38%, 27%, 27%, and 9%-63%, respectively. Safety endpoints using monotherapy–ICI, dual therapy–ICI, chemoradiation–ICI, radiotherapy–ICI, and chemo–ICI showed a failure to undergo resection in 0%-17%, 19%-33%, 8%, 13%, and 0%-46%, respectively. The trAEs grade ≥3 rates were 0%-20%, 10%-33%, 7%, 23%, and 0%-67%, respectively.

**Conclusion:**

In patients with resectable NSCLC stage, neoadjuvant immunotherapy can improve pathological response rates with acceptable toxicity. Further research is needed to identify patients who may benefit most from this approach, and adequately powered trials to establish clinically meaningful benefits are awaited.

## Highlights

• Neoadjuvant strategies involving immunotherapy are currently being investigated in NSCLC.
• Neoadjuvant immunotherapy was associated with improvement in pathological response rates.
• Neoadjuvant immunotherapy can be safe and feasible with acceptable surgical delay and trAEs.
• Further research is needed to identify patients benefiting most from these approaches.

## Introduction

In early and locally advanced stage non-small-cell lung cancer (NSCLC), surgery is the cornerstone of curative-intent treatments, resulting in a 5-year overall survival rate varying from 92% in stage IA to 26% in stage IIIB.^1^ However, despite a complete resection, ∼30%-55% of patients subsequently develop disease recurrence, mainly at distant sites.^2^^,^^3^ The addition of neoadjuvant or adjuvant chemotherapy resulted in an absolute improvement of only 5% in the 5-year survival.^4^

Recently, immune checkpoint inhibitors (ICIs), a class of immune oncology drugs, have proven their efficacy in the treatment of advanced stage NSCLC. The observed response rates in stage IV NSCLC have paved the way for multiple phase I-II trials that investigated the value of ICIs in a neoadjuvant setting for resectable (stage I-IIIA) or potentially resectable (stage IIIB) lung cancer. In this context, several ICIs have been explored; either as monotherapy or in combination with other treatment modalities such as a second ICI, radiotherapy, or chemotherapy. Although results are promising thus far, significant toxicities have also been reported.^5^, ^6^, ^7^ Nevertheless, there are several considerations that support the use of neoadjuvant ICIs, for example, tumor-specific antigens may prime the immune system, leading to sustained antitumor T-cell immune responses and benefits in long-term tumor control.^8^

This systematic review aims to provide an overview of the reported data on the efficacy and safety of neoadjuvant ICIs in patients with resectable or potentially resectable NSCLCs.

## Methods

This systematic review was reported according to the Preferred Reporting Items for Systematic Reviews and Meta-analysis (PRISMA) statement.^9^ The protocol for this study is registered in PROSPERO with registration number CRD42021235759.

### Search strategy

A systematic review of the literature was carried out by searching MEDLINE (PubMed) and Embase libraries. Published data of completed trials assessing the efficacy and safety of neoadjuvant immunotherapy in resectable NSCLCs were identified. In addition, conference abstracts with preliminary results of clinical trials were explored. Search terms included ‘non-small cell lung cancer’, ‘neoadjuvant’, and ‘immunotherapy’ for which Medical Subject Headings (MeSH Terms), Emtree terms, and/or free text words were identified. A detailed overview of the search strategy is provided in Supplementary Tables S1 and S2, available at https://doi.org/10.1016/j.esmoop.2021.100244. Ultimately, the final search was conducted on 16 July 2021.

### Study inclusion and exclusion criteria

Inclusion criteria consisted of clinical trials including patients (i) with pathologically diagnosed NSCLC presenting in a potentially resectable, nonmetastatic stage (I-IIIB); (ii) receiving planned ICIs in a neoadjuvant setting prior to surgical resection, and (iii) with data available on efficacy and safety. Exclusion criteria comprised studies addressing patients (i) with pathologically diagnosed NSCLC in an unresectable and metastatic stage, (ii) receiving immunotherapies other than ICIs, and (iii) with absence of reported data on the efficacy and safety of ICI treatment. The following types of reports were excluded: (i) articles reporting on duplicate results, (ii) study protocols, (iii) (systematic) reviews, (iv) editorials, (v) case reports, (vi) comments, (vii) articles in languages other than English, (viii) retrospective studies, (ix) studies missing data on >2 of 5 required endpoints, (x) case series with *n* < 5 receiving immunotherapy, (xi) studies providing multiple ICI regimens within a single study arm, and (xii) meta-analyses.

### Primary outcome and definitions

The primary outcome was efficacy and safety of neoadjuvant immunotherapy in resectable NSCLCs. The efficacy was defined by either major pathological response (MPR) or pathological complete response (pCR). Radiological responses assessed by RECIST version 1.1 were not evaluated. Safety was defined as (i) rate of failure to undergo resection, (ii) surgical delay (as defined by the authors of the included studies), and (iii) the incidence of treatment-related adverse events (trAEs) grade ≥3 according to the National Cancer Institute Common Terminology Criteria for Adverse Events.

## Results

### Study screening and selection

**Figure 1:**
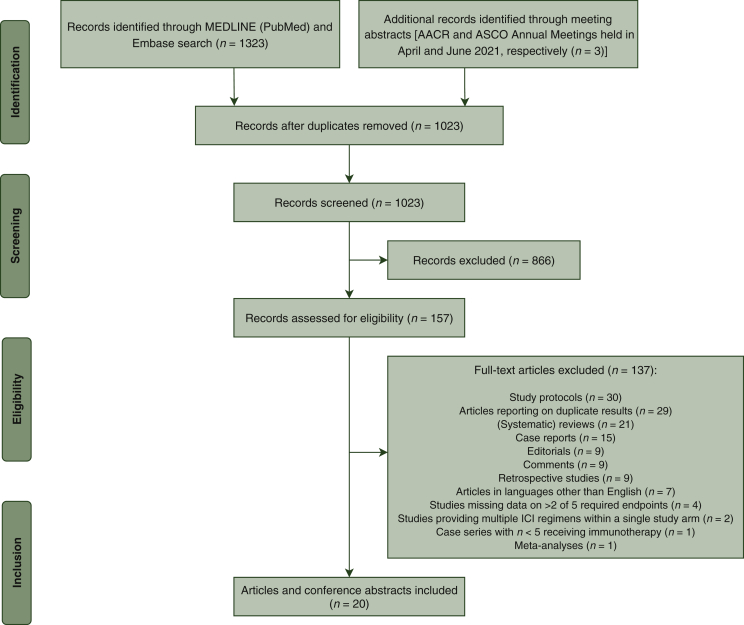
PRISMA flowchart. AACR, American Association for Cancer Research; ASCO, American Society of Clinical Oncology; PRISMA, Preferred Reporting Items for Systematic Reviews and Meta-analysis.

The literature search identified a total of 1323 records, with three additional records identified through searching the meeting abstracts of the American Association for Cancer Research (AACR) and American Society of Clinical Oncology (ASCO) Annual Meetings held in April and June 2021, respectively.^10^, ^11^, ^12^ A total of 303 records were excluded by deduplication, resulting in a final number of 1023 records. All records were independently screened by two authors (EBU and CD) to identify eligible studies based on the predefined inclusion and exclusion criteria. Differences in interpretation between the two reviewers were discussed to reach consensus and make a final decision on eligibility. From the 1023 records, 866 were excluded based on title and abstract and another 137 due to ineligibility, leaving 20 articles with a total of 1066 patients for inclusion. These 20 articles represent 15 single-arm cohort studies^11^^,^^13^, ^14^, ^15^, ^16^, ^17^, ^18^, ^19^, ^20^, ^21^, ^22^, ^23^, ^24^, ^25^, ^26^ and four randomized studies with two arms.^10^^,^^12^^,^^27^, ^28^, ^29^ The PRISMA flowchart depicting study selection is presented in Figure 1. An overview of the study characteristics is presented in Table 1. An outline of the excluded clinical studies is provided in Supplementary Table S3, available at https://doi.org/10.1016/j.esmoop.2021.100244.

**Table 1:** Study characteristics

Study	Phase	Study design	*N*	Stage I/II/III%^a^	ICI	Dose	Primary endpoints
Monotherapy–ICI							
Tong et al.^25^	II	Single-arm cohort study	30	30/43/27	Pembrolizumab	Two cycles 200 mg IV Q3W	Safety, feasibility
Eichhorn et al.^14^	II	Single-arm cohort study	15	0/40/60	Pembrolizumab	Two cycles 200 mg IV Q3W	Safety, feasibility
Lee et al.^18^	II	Single-arm cohort study	181	9/41/49	Atezolizumab	Two cycles 1200 mg IV Q3W	MPR
Gao et al.^16^	Ib	Single-arm cohort study	40	20/35/45	Sintilimab	Two cycles 200 mg IV Q3W	Safety
Besse et al.^13^	II	Single-arm cohort study	30	50/20/30	Atezolizumab	One cycle 1200 mg IV Q3-4W	% of patients without major toxicities or morbidities
Forde et al.^15^	II	Single-arm cohort study	22	19/48/33	Nivolumab	Two cycles 3 mg/kg IV Q2W	Safety, feasibility
Dual–ICI							
Cascone et al.^27^	II	Randomized two-arm study	23	48/30/22	Nivolumab	Three cycles 3 mg/kg IV Q2W	MPR
			21	57/24/19	Nivolumab Ipilimumab	Three cycles 3 mg/kg IV Q2W One cycle 1 mg/kg IV Q6W	
Reuss et al.^20^	II	Single-arm cohort study	9^b^	11/22/67	Nivolumab	Three cycles 3 mg/kg IV Q2W	Safety
					Ipilimumab	One cycle 1 mg/kg IV Q6W	
Chemotherapy with ICI							
Rothschild et al.^21^	II	Single-arm cohort study	67	0/0/100	Durvalumab	Two cycles 750 mg IV Q2W	EFS at 12 months
Zhao et al.^11^	II	Single-arm cohort study	33	0/0/100	Toripalimab	Three cycles 240 mg Q3W	MPR
Forde et al.^10^ and Spicer et al.^12^	III	Randomized two-arm study	179	23/14/63	Nivolumab	Three cycles 360 mg IV Q3W	pCR, EFS
			179	22/13/64	Chemo alone arm		
Shen et al.^22^	—	Single-arm cohort study	37	0/8/92	Pembrolizumab	Two cycles 2 mg/kg IV Q3W	pCR
Lei et al.^29^	II	Randomized two-arm study	7	0/0/100	Camrelizumab	Three cycles 200 mg IV Q3W	pCR
			7	0/0/100	Chemo alone arm		
Shu et al.^23^	II	Single-arm cohort study	30	0/23/77	Atezolizumab	Four cycles 1200 mg IV Q3W	MPR
Provencio et al.^19^	II	Single-arm cohort study	46	0/0/100	Nivolumab	Three cycles 360 mg IV Q3W	PFS at 24 months
Tfayli et al.^24^	II	Single-arm cohort study	15^c^	13/33/54	Avelumab	Four cycles 10 mg/kg IV Q2W	ORR by RECIST version 1.1
Yang et al.^26^	II	Single-arm cohort study	24	0/21/79	Ipilimumab	Two cycles 10 mg/kg IV	Safety, feasibility
Chemoradiotherapy with ICI							
Hong et al.^17^	I/II	Single-arm cohort study	11	0/0/100	Durvalumab	Two cycles 1500 mg IV Q4W	pCR
Radiotherapy with ICI							
Altorki et al.^28^	II	Randomized two-arm study	30	37/16/47	Durvalumab	Two cycles 1120 mg IV Q3W	MPR
			30	26/33/40	Durvalumab + SBRT		

EFS, event-free survival; ICI, immune checkpoint inhibitor; IV, intravenous; MPR, major pathological response; *N*, number of patients included; ORR, objective response rate; pCR, pathological complete response; PFS, progression-free survival; RECIST, response evaluation criteria in solid tumors; SBRT, stereotactic body radiotherapy.

a Either the 7th or 8th edition of TNM staging was assessed in the included studies to determine the stages of disease.

b Study was terminated by investigator consensus due to toxicities.

c Study was terminated due to failure to achieve required response rate.

### Efficacy

**Figure 2:**
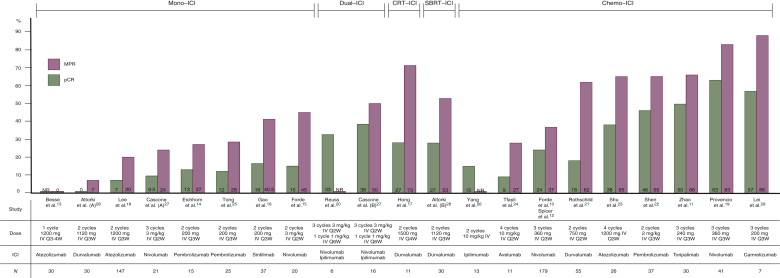
Graphical abstract showing the efficacy outcome measurements (MPR, pCR). Altorki et al. (A) = arm with patients receiving durvalumab monotherapy; Altorki et al. (B) = arm with patients receiving durvalumab + SBRT; Cascone et al. (A) = arm with patients receiving nivolumab monotherapy; Cascone et al. (B) = arm with patients receiving nivolumab + ipilimumab. Studies were ranked according to the MPR rate. Abbreviations: CRT, chemoradiotherapy; chemo, chemotherapy; ICI, immune checkpoint inhibitor; mono, monotherapy; IV, intravenous; MPR, major pathological response; *N*, number of resected tumors; NR, not reported; pCR, pathological complete response; SBRT, stereotactic body radiotherapy.

The efficacy outcome measurements are presented in Table 2. The results are graphically summarized in Figure 2.

**Table 2:** Efficacy outcome measurements

Study	Study arm	MPR	pCR
Monotherapy–ICI			
Range over all studies		0%-45%	7%-16%
Tong et al.^25^, *n* (%)		7/25 (28)	3/25 (12)
Eichhorn et al.^14^, *n* (%)		4/15 (27)	2/15 (13)
Lee et al.^18^, *n* (%)		30/147 (20)^a^	10/147 (7)^a^
Gao et al.^16^, *n* (%)		15/37 (40.5)	6/37 (16)
Besse et al.^13^, *n* (%)		0/30 (0)	NR
Forde et al.^15^, *n* (%)		9/20 (45)	3/20 (15)
Dual–ICI			
Cascone et al.^27^, *n* (%)	Nivo Ipi/nivo	5/21 (24) 8/16 (50)	2/21 (9.5) 6/16 (38)
Reuss et al.^20^, *n* (%)		NR	2/6 (33)
Chemotherapy with ICI			
Range over all studies		27%-86%	9%-63%
Rothschild et al.^21^, *n* (%)		34/55 (62)	10/55 (18)
Zhao et al.^11^, *n* (%)		20/30 (66)	15/30 (50)
Forde et al.^10^ and Spicer et al.^12^, *n* (%)	Nivo + chemo Chemo alone	66/179 (37) 4/179 (2)	43/179 (24) 16/179 (9)
Shen et al.^22^, *n* (%)		24/37 (65)	17/37 (46)
Lei et al.^29^, *n* (%)	Cam + chemo	6/7 (86)	4/7 (57)
	Chemo alone	2/6 (33)	1/6 (17)
Shu et al.^23^, *n* (%)		17/26 (65)	10/26 (38)
Provencio et al.^19^, *n* (%)		34/41 (83)	26/41 (63)
Tfayli et al.^24^, *n* (%)		3/11 (27)	1/11 (9)
Yang et al.^26^, *n* (%)		NR	2/13 (15)
Chemoradiotherapy with ICI			
Hong et al.^17^, *n* (%)		8/11 (73)	3/11 (27)
Radiotherapy with ICI			
Altorki et al.^28^, *n* (%)	Durva Durva + SBRT	2/30 (7) 16/30 (53)	0/30 (0) 8/30 (27)

Cam, camrelizumab; Chemo, chemotherapy; Durva, durvalumab; ICI, immune checkpoint inhibitor; Ipi/nivo, ipilimumab/nivolumab; MPR, major pathological response; Nivo, nivolumab; NR, not reported; pCR, pathological complete response; SBRT, stereotactic body radiotherapy.

a Patients with oncogenic driver mutations were excluded from pathological evaluations.

#### Major pathological response (MPR)

MPR, defined as ≤10% viable tumor tissue on pathological examination,^30^ was reported in 17 of 19 studies included. Two studies did not report on MPR.^20^^,^^26^ In the monotherapy–ICI group, MPR rates up to 45% were reported,^15^ and one study reported absence of MPR in the included patients.^13^ The addition of ipilimumab to nivolumab led to a 50% MPR rate in one study.^27^ Regarding the combination of chemoradiotherapy with durvalumab, MPR rate was reported to be 73%.^17^ The chemotherapy–ICI group shows higher MPR rates when compared with the monotherapy–ICI group, with rates varying from 27%^24^ to 86%.^29^ Radiotherapy combined with durvalumab led to MPR in 53% of patients.^28^

#### Pathological complete response (pCR)

pCR, which is defined as the absence of residual viable tumor cells after induction therapy, was reported in all studies except one.^13^ The monotherapy–ICI group shows consistent rates of pCR ranging from 0%^28^ to 16%,^16^ with one study reporting a pCR rate for half the patients achieving MPR.^14^ The addition of ipilimumab to nivolumab led to an absolute increase of pCR, with reported rates of 33% and 38%.^20^^,^^27^ Combining chemoradiotherapy with durvalumab led to pCR rate of 27%.^17^ The chemotherapy–ICI group reported rates varying from 9%^24^ to 63%,^19^ with most studies reporting pCR in more than half of the patients achieving MPR.^10^^,^^11^^,^^19^^,^^22^^,^^23^^,^^29^ When neoadjuvant durvalumab was combined with radiotherapy, 27% of the included patients achieved pCR.^28^

### Safety

**Figure 3:**
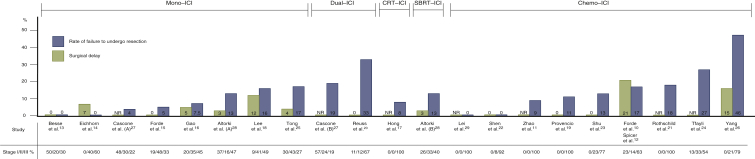
Graphical abstract showing safety outcome measurements (rate of failure to undergo resection, surgical delay). Altorki et al. (A) = arm with patients receiving durvalumab monotherapy; Altorki et al. (B) = arm with patients receiving durvalumab + SBRT; Cascone et al. (A) = arm with patients receiving nivolumab monotherapy; Cascone et al. (B) = arm with patients receiving nivolumab + ipilimumab. Studies were ranked according to the rate of failure to undergo resection. Abbreviations: chemo, chemotherapy; CRT, chemoradiotherapy; ICI, immune checkpoint inhibitor; mono, monotherapy; NR, not reported; SBRT, stereotactic body radiotherapy.

The safety outcome measurements are presented in Table 3. The results are graphically summarized in Figure 3.

**Table 3:** Safety outcome measurements

Study	Study arm	Failure to undergo resection	Surgical delay	trAEs grade ≥3
Monotherapy–ICI				
Range over all studies		0%-17%	0%-12%	0%-20%
Tong et al.^25^, *n* (%)		5/30 (17)	1/25 (4)	1/30 (3)
Eichhorn et al.^14^, *n* (%)		0/15 (0)	1/15 (7)^a^	3/15 (20)
Lee et al.^18^, *n* (%)		29/181 (16)	19/159 (12)^b^	9/181 (5)^f^
Gao et al.^16^, *n* (%)		3/40 (7.5)	2/37 (5)	4/40 (10)
Besse et al.^13^, *n* (%)		0/30 (0)	0/30 (0)^c^	0/30 (0)
Forde et al.^15^, *n* (%)		1/21 (5)	0/20 (0)	1/22 (4.5)
Dual–ICI				
Cascone et al.^27^, *n* (%)	Nivo	1/23 (4)	8/37 (22)^d^	3/23 (13)
	Ipi/nivo	4/21 (19)		2/21 (10)
Reuss et al.^20^, *n* (%)		3/9 (33)	0/6 (0)	3/9 (33)
Chemotherapy with ICI				
Range over all studies		0%-46%	0%-21%	0%-67%
Rothschild et al.^21^, *n* (%)		12/67 (18)	NR	45/67 (67)^j^ 8/62 (13)^k^
Zhao et al.^11^, *n* (%)		3/33 (9)	NR	3/33 (9)
Forde et al.^10^ and Spicer et al.^12^, *n* (%)	Nivo + chemo	30/179 (17)	31/149 (21)	60/179 (33.5)
	Chemo alone	44/179 (25)	24/135 (18)	66/179 (37)
Shen et al.^22^, *n* (%)		0/37 (0)	0/37 (0)	4/37 (11)
Lei et al.^29^, *n* (%)	Cam + chemo	0/7 (0)	NR	NR
	Chemo alone	0/6 (0)^g^	NR	NR
Shu et al.^23^, *n* (%)		4/30 (13)	0/26 (0)	15/30 (50)
Provencio et al.^19^, *n* (%)		5/46 (11)	0/41 (0)	14/46 (30)
Tfayli et al.^24^, *n* (%)		4/15 (27)	NR	4/15 (27)
Yang et al.^26^, *n* (%)		11/24 (46)	2/13 (15)^e^	11/24 (46)
Chemoradiotherapy with ICI				
Hong et al.^17^, *n* (%)		1/12 (8)^g^	NR	1/14 (7)
Radiotherapy with ICI				
Altorki et al.^28^, *n* (%)	Durva Durva + SBRT	4/30 (13) 4/30 (13)	1/30 (3)^h^ 1/30 (3)^i^	6/30 (20) 7/30 (23)

Cam, camrelizumab; Chemo, chemotherapy; Durva, durvalumab; ICI, immune checkpoint inhibitor; Ipi/nivo, ipilimumab/nivolumab; Nivo, nivolumab; NR, not reported; SBRT, stereotactic body radiotherapy; trAEs, treatment-related adverse events.

a Incidence of surgical delay of 13 days.

b Incidence of surgical delay outside the ±10-day window.

c Incidence of surgical delay >15 days.

d Incidence of surgical delay > 42 days; no distinction between the two arms was made.

e Incidence of surgical delay ≥28 days.

f Postoperative trAEs grade ≥3 excluded.

g Patients awaiting surgery are excluded.

h Incidence of surgical delay of 4 weeks.

i Incidence of surgical delay of 7 weeks.

j trAEs grade ≥3 during neoadjuvant chemotherapy.

k trAEs grade ≥3 during neoadjuvant immunotherapy.

#### Failure to undergo resection

The rates of failure to undergo resection varied from 0%^13^^,^^14^^,^^22^^,^^29^ to 46%.^26^ There was no difference in these rates when considering the different groups (monotherapy–ICI, dual–ICI, chemoradiotherapy–ICI, radiotherapy–ICI, chemotherapy–ICI). There were various reasons reported for not proceeding to a resection: disease progression,^10^^,^^11^^,^^18^, ^19^, ^20^, ^21^^,^^23^, ^24^, ^25^, ^26^, ^27^, ^28^ inadequate lung function,^24^^,^^27^ persistent N2 disease,^26^ unresectable disease,^21^^,^^23^^,^^25^ trAEs,^10^^,^^27^ location of tumor,^15^^,^^26^ or patient refusal.^11^ One study did not report on the specific reason for a patient not to proceed to the planned surgery.^16^

#### Surgical delay

Surgical delays were reported using author-specified definitions varying from surgical delay outside the ±10-day window to a surgical delay of 7 weeks. The rates of patients experiencing surgical delay ranged from 0%^13^ to 22%,^27^ with an outlier study reporting a pooled incidence for the two arms. No surgical delay was reported in 6 of the 19 included studies.^13^^,^^15^^,^^19^^,^^20^^,^^22^^,^^23^ Reported surgical delays were most often attributed to trAEs.^14^^,^^16^^,^^25^^,^^26^^,^^28^ Three studies did not disclose any details on the reasons leading to the reported surgical delays,^13^^,^^18^^,^^27^ and another five did not report any data on surgical delay.^11^^,^^17^^,^^21^^,^^24^^,^^29^

#### Treatment-related adverse events (trAEs) grade ≥3

The monotherapy–ICI group reported rates of trAEs grade ≥3 up to 20%,^14^ with one study reporting no trAEs grade ≥3 at all.^13^ Most of these trAEs were pulmonary related (i.e. pneumonitis or pneumonia).^15^^,^^16^^,^^18^^,^^27^ In the chemotherapy–ICI group, reported rates ranged from 0%^29^ to 67%.^21^ Here, the reported trAEs were attributed to hematological disorders,^19^^,^^22^, ^23^, ^24^ ipilimumab-related diarrhea,^26^ and chemotherapy-related toxicities.^26^ The combination of chemoradiation with durvalumab led to one grade 3 trAE due to neutropenia.^17^ The dual–ICI group reported trAEs grade ≥3 rates of 10%^27^ and 33%,^20^ with study closure of the latter trial due to the observed toxicity rate. Regarding the combination of durvalumab with radiotherapy, the most frequent reported trAEs included grade 3 hyperlipasaemia.^28^ One study did not report on adverse events related to the neoadjuvant regimen.^29^

## Discussion

This systematic review on neoadjuvant use of ICIs in resectable NSCLC summarizes the efficacy and safety outcomes of the currently published trials. The results suggest that this approach is safe and feasible, with acceptable surgical delays and grade ≥3 trAEs.

MPR, defined as the presence of ≤10% residual vital tumor cells in the resection specimen, serves as a major primary endpoint in many neoadjuvant immunotherapy studies. MPR is proposed as a surrogate endpoint for survival, as it has been associated with improved survival,^31^ and could therefore provide a faster means of comparing different neoadjuvant treatment regimens, and shorten the period needed to evaluate neoadjuvant therapies.^32^ A major limitation of using MPR is the lack of precision due to the inherent interobserver variability. Weissferdt et al.^32^ reported on a prospective study in 151 NSCLC patients treated with neoadjuvant chemotherapy, where two trained pathologists separately scored the residual tumor cells percentage in the resected specimens. The authors reported that the MPR assessed by both pathologists was associated with long-term overall survival in patients with NSCLC undergoing neoadjuvant chemotherapy (hazard ratio 2.68; *P* = 0.01). The levels of agreement between the two pathologists were high (*R*^2^ = 0.994), and it was proposed that at least three slides should be read to accurately determine MPR.

In the published trials, MPR rates ranged from between 0% and 45% of patients in the monotherapy–ICI cohort, 50% for dual–ICI cohort, 73% for the chemoradiation–ICI cohort, 53% for the radiotherapy–ICI cohort, and 27%-86% in the chemotherapy–ICI cohort. These MPR rates are higher as compared with those after preoperative chemotherapy alone, which was reported to be ∼16%.^33^ The synergistic effect of chemotherapy and ICIs, with the cytotoxic chemotherapy functioning as a sensitizer for immune checkpoint blockade, might explain the high rates of MPR in this group.^34^ Sixteen of 19 included studies reported on pCR, with rates ranging between 12% and 63%, which are higher than the 10.5% reported after neoadjuvant chemotherapy.^35^

Some aspects of treatment efficacy merit further discussion. First, although high rates of pathological responses by MPR and pCR were reported in most included studies, it is still unclear as to which stages of NSCLC benefit the most from neoadjuvant therapy. A stage-based assessment of pathological responses is important as it may allow for improved design of future trials in specific disease stages.^30^ Second, responses to neoadjuvant ICIs can be influenced by the tumor molecular contexture, as several genomic alterations such as STK11 mutations have been associated with resistance to anti-PD-(L)1 agents.^36^ Such patient subgroups may be less likely to respond to ICIs, while suffering the risks of immune-related AEs. By contrast, biomarkers which have been associated with responses to ICIs include PD-L1 expression in stromal cells,^16^ CD28 expression on CD8^+^ tumor-infiltrating lymphocytes (TILs),^37^ and stromal TILs.^38^ This highlights the need to identify appropriate predictive biomarkers for selecting patients upfront. Third, the lack of a standardized approach for pathological reporting of resected lung carcinomas following neoadjuvant treatment may reflect a lack of sufficient input from pathologists in study design.^39^ The ‘IASLC Multidisciplinary Recommendations for Pathologic Assessment of Lung Cancer Resection Specimens After Neoadjuvant Therapy’ were published in 2020, and can serve to standardize reporting on the pathological outcomes.^30^

Rates of failure to undergo resection were generally acceptable, although higher rates of unresected patients were reported in studies that included more patients with stage III NSCLC, and disease progression was the main reason accounting for not undergoing a resection. However, it is questionable if the decision to not proceed with surgery in patients who developed distant metastases during induction therapy can be labeled therapy failure as it is likely that these patients may not have benefited from a resection in the first place.

The surgical delays were within acceptable ranges in all studies, with 6 of 19 studies reporting no surgical delays at all. The grade ≥3 treatment-related AEs were similar to those reported previously for chemo–ICI regimens.^40^^,^^41^ However, the reported trAE rates in most included studies were lower compared with preoperative chemotherapy.^42^

Limitations of the current systematic review include the substantial heterogeneity in the included trials, some of which included only a few patients. In addition, most were single-arm cohort studies, and no randomized controlled trials were included. Consequently, there is a risk of biases which limit the ability to draw reliable conclusions. Moreover, this systematic review included studies reporting only preliminary results of clinical trials, and a publication bias cannot be ruled. Our conclusions may therefore require revision once the final analyses of study data are presented. Another limitation is the fact that this systematic review assessed only two indicators for the efficacy outcome measurements, and three indicators for the safety outcome measurements. We did not report on the radiological response rates using RECIST version 1.1, as radiological assessments after neoadjuvant therapies have a poor correlation with the pathological response and survival.^43^

Currently, numerous phase II/III clinical trials are underway to further investigate the efficacy of neoadjuvant immunotherapy in resectable NSCLC, and to compare these approaches with placebo or standard treatments. CheckMate 816 was the first phase III randomized clinical trial that reported on pathological response and toxicity of neoadjuvant nivolumab/chemotherapy versus chemotherapy alone. This trial included 358 resectable stage IB-IIIA NSCLC patients, and confirmed the findings from previous phase II trials, showing a 21.6% increase in pCR (by blinded independent central review) in the nivolumab arm and no differences in safety outcomes.^10^ Supplementary Table S4, available at https://doi.org/10.1016/j.esmoop.2021.100244 provides an overview of the major ongoing trials.

In conclusion, the currently published data suggest that neoadjuvant ICIs can be considered safe and feasible, with encouraging pathological responses. Further research is needed to identify patients who may benefit most from this approach, and adequately powered trials to establish clinically meaningful benefits are awaited.
