# Supplementary material for: Neoadjuvant immune checkpoint inhibitors in resectable non-small-cell lung cancer: a systematic review
Source: ESMO Open. 2021 Aug 31;6(5):100244. doi: 10.1016/j.esmoop.2021.100244 (PMC8414043; doi:10.1016/j.esmoop.2021.100244)
Supplement: Supplementary Tables S1-S4 [file mmc1.docx]

**Supplementary**

**Table 1. MEDLINE (PubMed) search**

| **Search** | **Query** | **Items found** |
| --- | --- | --- |
| #4 | #1 AND #2 AND #3 | 311 |
| #3 | "Neoadjuvant Therapy"[MeSH] OR neoadjuvant[tiab] OR "neo-adjuvant"[tiab] OR perioperative[tiab] OR preoperative[tiab] OR "peri-operative"[tiab] OR "pre-operative"[tiab] | 417.985 |
| #2 | Immunotherapy[MeSH] OR "Antibodies, Monoclonal, Humanized"[MeSH] OR "Programmed Cell Death 1 Receptor"[MeSH] OR "CTLA-4 Antigen"[MeSH] OR "B7-H1 Antigen"[MeSH] OR "Immune Checkpoint Inhibitors"[MeSH] OR immunotherap*[tiab] OR immuno-therap*[tiab] OR immunetherap*[tiab] OR immune-therap*[tiab] OR immune checkpoint inhibit*[tiab] OR nivolumab[tiab] OR ipilimumab[tiab] OR sintilimab[tiab] OR durvalumab[tiab] OR atezolizumab[tiab] OR pembrolizumab[tiab] OR avelumab[tiab] OR tremelimumab[tiab] OR camrelizumab[tiab] OR tislelizumab[tiab] OR toripalimab[tiab] | 433.102 |
| #1 | "Carcinoma, Non-Small-Cell Lung"[MeSH] OR non-small cell lung cancer*[tiab] OR non-small cell lung carcinoma*[tiab] OR nonsmall cell lung cancer*[tiab] OR nonsmall cell lung carcinoma*[tiab] OR NSCLC[tiab] | 84.838 |
| **Abbreviations:** [MeSH] = Medical Subject Headings; [tiab] = Title/Abstract | |  |

**Table 2. EMBASE search**

| **Search** | **Query** | **Items found** |
| --- | --- | --- |
| #4 | #1 AND #2 AND #3 | 1012 |
| #3 | 'neoadjuvant therapy'/exp OR ‘neoadjuvant’:ab,ti,kw OR 'neo-adjuvant':ab,ti,kw OR ‘perioperative’:ab,ti,kw OR ‘preoperative’:ab,ti,kw OR 'peri-operative':ab,ti,kw OR 'pre-operative':ab,ti,kw | 607.297 |
| #2 | 'immunotherapy'/exp OR 'monoclonal antibody'/exp OR 'immune checkpoint inhibitor'/exp OR 'programmed death 1 receptor'/exp OR 'programmed death 1 ligand 1'/exp OR 'cytotoxic t lymphocyte antigen 4'/exp OR ‘immunotherap*’:ab,ti,kw OR 'immuno-therap*':ab,ti,kw OR ‘immunetherap*’:ab,ti,kw OR 'immune-therap*':ab,ti,kw OR 'immune checkpoint inhibit*':ab,ti,kw OR ‘nivolumab’:ab,ti,kw OR ‘ipilimumab’:ab,ti,kw OR ‘sintilimab’:ab,ti,kw OR ‘durvalumab’:ab,ti,kw OR ‘atezolizumab’:ab,ti,kw OR ‘pembrolizumab’:ab,ti,kw OR ‘avelumab’:ab,ti,kw OR ‘tremelimumab’:ab,ti,kw OR ‘camrelizumab’:ab,ti,kw OR ‘tislelizumab’:ab,ti,kw OR ‘toripalimab’:ab,ti,kw | 889.655 |
| #1 | 'non small cell lung cancer'/exp OR 'non-small cell lung cancer*':ab,ti,kw OR 'non-small cell lung carcinoma*':ab,ti,kw OR 'nonsmall cell lung cancer*':ab,ti,kw OR 'nonsmall cell lung carcinoma*':ab,ti,kw OR ‘NSCLC’:ab,ti,kw | 188.216 |

**Abbreviations:** /exp = explosion in Emtree terms; :ti,ab,kw = title, abstract, keywords

**Table 3. Excluded clinical studies**

|  | Phase | Study design | N | Stage I/II/III % | ICI | Primary endpoints | Efficacy | | Safety | | |
| --- | --- | --- | --- | --- | --- | --- | --- | --- | --- | --- | --- |
|  |  |  |  |  |  |  |  |  |  |  |  |
|  |  |  |  |  |  |  | MPR | pCR | Unperformed resections | Surgical delay | trAEs ≥ grade 3 |
| **Monotherapy ICI** |  |  |  |  |  |  |  |  |  |  |  |
|  |  |  |  |  |  |  |  |  |  |  |  |
| Wislez, 2020^1^ | II | Single-arm cohort study | 50 | 11/87/2 | Durvalumab | % R0 resections | NR | NR | NR | NR | None |
| Bar, 2019^2^ | I | Single-arm cohort study | 10 | NR | Pembrolizumab | Safety, pathological and radiological response | 4/10 (40%) | NR | NR | None | NR |
| Ready, 2019^3^ | II | Single-arm cohort study | 30 | 27/47/27 | Pembrolizumab | Surgical tolerability | NR | NR | 5/30 (17%) | NR | NR |
| **Chemotherapy with ICI** | |  |  |  |  |  |  |  |  |  |  |
|  |  |  |  |  |  |  |  |  |  |  |  |
| Duan, 2021^4^ | - | Single-arm study | 23 | 0/26/64 | Nivolumab/sintilimab/pembrolizumab | Safety, efficacy | 10/20 (50%) | 6/20 (30%) | 3/23 (13%) | NR | NR |
| Chen, 2020^5^ | - | Non-randomized 2-arm study | 9 | 0/56/44 | Pembrolizumab | Feasibility, safety | 4/4 (100%) | 3/4 (75%) | NR | None | None |
| **Abbreviations:** ICI = Immune Checkpoint Inhibitor; DFS = Disease Free Survival; trAEs = treatment-related Adverse Events; MPR = Major Pathological Response; pCR = pathological Complete Response; NR = Not Reported | | | | | | | | | | | |

**Table 4. Ongoing trials**

| **Study** | **Study acronym** | **Phase** | **Study design** | **Estimated N** | **Stage** | **ICI** | **Primary endpoints** |
| --- | --- | --- | --- | --- | --- | --- | --- |
| **Monotherapy ICI** |  |  |  |  |  |  |  |
|  |  |  |  |  |  |  |  |
| Kagimoto, 2021^6^ | POTENTIAL | II | Single-arm study | 50 | I | Nivolumab | pCR |
| Lee, 2020^7^ | CANOPY-N | II | Randomized 3-arm study | 110 | IB-IIIA | Pembrolizumab | MPR |
| Tsuboi, 2020^8^ | KEYNOTE-671 | III | Randomized 2-arm study | 786 | II-IIIB | Pembrolizumab | EFS, OS |
| Campelo, 2019^9^ | NeoCOAST | II | Randomized 4-arm study | 80 | I-IIIA | Durvalumab | MPR |
| **Chemotherapy with ICI** |  |  |  |  |  |  |  |
|  |  |  |  |  |  |  |  |
| Ma, 2021^10^ | - | II | Single-arm study | 40 | IIIA | Sintilimab | DFS |
| Cascone, 2020^11^ | Checkmate-77T | III | Randomized 2-arm study | 452 | IIA-IIIB | Nivolumab | EFS |
| Peters, 2019^12^ | IMpower030 | III | Randomized 2-arm study | 450 | II-IIIB | Atezolizumab | MPR, EFS |
| Heymach, 2019^13^ | AEGEAN | III | Non-randomized 2-arm study | 800 | I-IIIA | Durvalumab | MPR, EFS |
| **Chemoradiotherapy with ICI** |  |  |  |  |  |  |  |
|  |  |  |  |  |  |  |  |
| Hamada, 2021^14^ | SQUAT | II | Single-arm study | 28 | IIIA-B | Durvalumab | MPR |
| Dickhoff, 2020^15^ | INCREASE | II | Single-arm study | 29 | IIB-IIIA | Nivolumab  Ipilimumab | Safety, pCR, MPR |
| **Radiotherapy with ICI** |  |  |  |  |  |  |  |
|  |  |  |  |  |  |  |  |
| Beal, 2021^16^ | - | II | Single-arm study | 30 | I-II | Nivolumab | pCR |
| **Abbreviations:** ICI = Immune Checkpoint inhibitor; EFS = Event Free Survival; PFS = Progression Free Survival; DFS = Disease Free Survival; MPR = Major Pathological Response; pCR = pathological Complete Response; OS = Overall Survival | | | | | | | |

**References**

1. Wislez M, Mazieres J, Lavole A, Zalcman G, Carre O, Egenod T, Caliandro R, Gervais R, Jeannin G, Molinier O, Massiani MA, Langlais A, Morin F, Le Pimpec Barthes F, Brouchet L, Assouad J, Milleron B, Damotte D, Antoine M, Westeel V. 1214O Neoadjuvant durvalumab in resectable non-small cell lung cancer (NSCLC): Preliminary results from a multicenter study (IFCT-1601 IONESCO). *Annals of Oncology*. 2020;31:S794. <https://doi.org/10.1016/j.annonc.2020.08.1416>

2. Bar J, Urban D, Ofek E, Ackerstein A, Redinsky I, Golan N, Kamer I, Simansky D, Onn A, Raskin S, Shulimzon T, Peled M, Zeitlin N, Halparin S, Jurkowicz M, Abukhalil R, Perelman M, Ben-Nun A. Neoadjuvant pembrolizumab (Pembro) for early stage non-small cell lung cancer (NSCLC): Updated report of a phase i study, MK3475-223. *Journal of Clinical Oncology*. 2019;37. <https://doi.org/10.1200/JCO.2019.37.15_suppl.8534>

3. Ready N, Tong B, Clarke J, Gu L, Wigle D, Dragnev K, Sporn T, Stinchcombe T, D’Amico T. P2.04-89 Neoadjuvant Pembrolizumab in Early Stage Non-Small Cell Lung Cancer (NSCLC): Toxicity, Efficacy, and Surgical Outcomes. *Journal of Thoracic Oncology*. 2019;14(10):S745. <https://doi.org/10.1016/j.jtho.2019.08.1594>

4. Duan H, Wang T, Luo Z, Tong L, Dong X, Zhang Y, Afzal MZ, Correale P, Liu H, Jiang T, Yan X. Neoadjuvant programmed cell death protein 1 inhibitors combined with chemotherapy in resectable non-small cell lung cancer: An open-label, multicenter, single-arm study. *Translational Lung Cancer Research*. 2021;10(2):1020-8. <https://doi.org/10.21037/tlcr-21-130>

5. Chen Y, Zhang L, Yan B, Zeng Z, Hui Z, Zhang R, Ren X, You J. Feasibility of sleeve lobectomy after neo-adjuvant chemo-immunotherapy in non-small cell lung cancer. *Transl Lung Cancer Res*. 2020;9(3):761-7. <https://doi.org/10.21037/tlcr-20-539>

6. Kagimoto A, Tsutani Y, Mimae T, Miyata Y, Ikeda N, Ito H, Maniwa Y, Suzuki K, Tsuboi M, Yoshimura K, Umemoto S, Okada M. Preoperative nivolumab to evaluate pathological response in patients with stage i non-small cell lung cancer: A study protocol of phase II trial (POTENTIAL). *BMJ Open*. 2021;11(3). <https://doi.org/10.1136/bmjopen-2020-043234>

7. Lee JM, Garrido P, Kim ES, Arslan C, Pujol JL, Song Y, Blin C, Rodrik-Outmezguine V, Mookerjee B, Passos V, Mok T. Randomized phase II study of canakinumab (CAN) or pembrolizumab (PEM) alone or incombination as neoadjuvant therapy in patients (Pts) with surgically resected (Stage IB-IIIA) non-small cell lungcancer (NSCLC): CANOPY-N. *Cancer Research*. 2020;80(16 SUPPL). <https://doi.org/10.1158/1538-7445.AM2020-CT192>

8. Tsuboi M, Luft A, Ursol G, Kato T, Levchenko E, Eigendorff E, Berard H, Zurawski B, Demedts I, Garassino MC, Yang J, Makarious K, Keller SM, Wakelee HA. Perioperative pembrolizumab + platinum-based chemotherapy for resectable locally advanced non-small cell lung cancer: The phase III KEYNOTE-671 study. *Annals of Oncology*. 2020;31:S801-S2. <https://doi.org/10.1016/j.annonc.2020.08.1437>

9. Campelo RG, Forde P, Weder W, Spicer J, He P, Hamid O, Martinez P, Cascone T. P2.04-28 NeoCOAST: Neoadjuvant Durvalumab Alone or with Novel Agents for Resectable, Early-Stage (I–IIIA) Non-Small Cell Lung Cancer. *Journal of Thoracic Oncology*. 2019;14(10):S719. <https://doi.org/10.1016/j.jtho.2019.08.1533>

10. Ma K, Sun C, Jia X, Wang X, Xu Y, Guo Y, Qiu S, Shao G, Zhang P, Liu Y, Ma X. P79.09 Neoadjuvant Combination Sintilimab plus Chemotherapy in Patients with Resectable Stage IIIA Non-Small Cell Lung Cancer. *Journal of Thoracic Oncology*. 2021;16(3):S649-S50. <https://doi.org/10.1016/j.jtho.2021.01.1188>

11. Cascone T, Provencio M, Sepesi B, Lu S, Aanur N, Li S, Spicer J. Checkmate 77T: A phase III trial of neoadjuvant nivolumab (NIVO) plus chemotherapy (chemo) followed by adjuvant nivo in resectable early-stage NSCLC. *Journal of Clinical Oncology*. 2020;38(15_suppl):TPS9076-TPS. <https://doi.org/10.1200/JCO.2020.38.15_suppl.TPS9076>

12. Peters S, Kim AW, Solomon B, Gandara DR, Dziadziuszko R, Brunelli A, Garassino MC, Reck M, Wang L, To I, Sun SW, Gitlitz BJ, Sandler A, Rizvi N. IMpower030: Phase III study evaluating neoadjuvant treatment of resectable stage II-IIIB non-small cell lung cancer (NSCLC) with atezolizumab (atezo) + chemotherapy. *Annals of Oncology*. 2019;30:ii30. <https://doi.org/10.1093/annonc/mdz064.014>

13. Heymach J, Taube J, Mitsudomi T, Harpole D, Aperghis M, Trani L, Powell M, Dennis P, Reck M. P1.18-02 The AEGEAN Phase 3 Trial of Neoadjuvant/Adjuvant Durvalumab in Patients with Resectable Stage II/III NSCLC. *Journal of Thoracic Oncology*. 2019;14(10):S625-S6. <https://doi.org/10.1016/j.jtho.2019.08.1318>

14. Hamada A, Soh J, Hata A, Nakamatsu K, Shimokawa M, Yatabe Y, Oizumi H, Tsuboi M, Horinouchi H, Yoshino I, Tanahashi M, Toyooka S, Okada M, Yokomise H, Yamashita M, Nishimura Y, Yamamoto N, Nakagawa K, Mitsudomi T. Phase II Study of Neoadjuvant Concurrent Chemo-immuno-radiation Therapy Followed by Surgery and Adjuvant Immunotherapy for Resectable Stage IIIA-B (Discrete N2) Non-small-cell Lung Cancer: SQUAT trial (WJOG 12119L). *Clin Lung Cancer*. 2021. <https://doi.org/10.1016/j.cllc.2021.04.006>

15. Dickhoff C, Senan S, Schneiders FL, Veltman J, Hashemi S, Daniels JMA, Fransen M, Heineman DJ, Radonic T, van de Ven PM, Bartelink IH, Meijboom LJ, Garcia-Vallejo JJ, Oprea-Lager DE, de Gruijl TD, Bahce I. Ipilimumab plus nivolumab and chemoradiotherapy followed by surgery in patients with resectable and borderline resectable T3-4N0-1 non-small cell lung cancer: the INCREASE trial. *BMC Cancer*. 2020;20(1):764. <https://doi.org/10.1186/s12885-020-07263-9>

16. Beal J, Gomes D, Taranto P, Koch L, Rezende AC, Samano M, Bibas B, Gomes O, Campregher P, Severino P, Marti L, Paes V, Chate R, Sales D, Schvartsman G. P82.02 Stereotactic Ablative Radiotherapy with Nivolumab for Early-Stage Operable Non-Small Cell Lung Cancer: a phase 2 study. *Journal of Thoracic Oncology*. 2021;16(3):S651-S2. <https://doi.org/10.1016/j.jtho.2021.01.1193>
